# Prostate Cancer Imaging and Biomarkers Guiding Safe Selection of Active Surveillance

**DOI:** 10.3389/fonc.2017.00256

**Published:** 2017-10-30

**Authors:** Zachary A. Glaser, Jennifer B. Gordetsky, Kristin K. Porter, Sooryanarayana Varambally, Soroush Rais-Bahrami

**Affiliations:** ^1^Department of Urology, University of Alabama at Birmingham, Birmingham, AL, United States; ^2^Department of Pathology, University of Alabama at Birmingham, Birmingham, AL, United States; ^3^Department of Radiology, University of Alabama at Birmingham, Birmingham, AL, United States

**Keywords:** prostatic adenocarcinoma, multiparametric MRI, cancer imaging, cancer genetics, cancer epigenetics

## Abstract

**Background:**

Active surveillance (AS) is a widely adopted strategy to monitor men with low-risk, localized prostate cancer (PCa). Current AS inclusion criteria may misclassify as many as one in four patients. The advent of multiparametric magnetic resonance imaging (mpMRI) and novel PCa biomarkers may offer improved risk stratification. We performed a review of recently published literature to characterize emerging evidence in support of these novel modalities.

**Methods:**

An English literature search was conducted on PubMed for available original investigations on localized PCa, AS, imaging, and biomarkers published within the past 3 years. Our Boolean criteria included the following terms: PCa, AS, imaging, biomarker, genetic, genomic, prospective, retrospective, and comparative. The bibliographies and diagnostic modalities of the identified studies were used to expand our search.

**Results:**

Our review identified 222 original studies. Our expanded search yielded 244 studies. Among these, 70 met our inclusion criteria. Evidence suggests mpMRI offers improved detection of clinically significant PCa, and MRI-fusion technology enhances the sensitivity of surveillance biopsies. Multiple studies demonstrate the promise of commercially available screening assays for prediction of AS failure, and several novel biomarkers show promise in this setting.

**Conclusion:**

In the era of AS for men with low-risk PCa, improved strategies for proper stratification are needed. mpMRI has dramatically enhanced the detection of clinically significant PCa. The advent of novel biomarkers for prediction of aggressive disease and AS failure has shown some initial promise, but further validation is warranted.

## Introduction

In 2017, there will be an estimated 161,000 new cases of prostate cancer (PCa) in the United States, representing approximately 20% of new cancer diagnoses and the third leading cause of death in men (1). Since the adoption of prostate specific antigen (PSA) screening in the 1990s, the incidence of PCa has significantly increased, while age-adjusted cancer-specific mortality has declined nearly 45% (2). This is largely attributed to the increased detection of localized, low-risk disease, and immediate intervention in these patients may not be necessary (2, 3). Furthermore, early intervention exposes patients to the potential side effects of medications, radiation and surgery, and may cause undue damage to quality of life (4). Since cancer-specific survival in this subset of patients is very high, even with conservative management, a less-invasive management approach may be more appropriate (5).

Close monitoring by active surveillance (AS), which includes serial PSAs, digital rectal exams and transrectal ultrasound (TRUS) biopsies, and more recently, MRI, has become a widely adopted strategy to monitor indolent disease (3–5). About 50% of men on AS will eventually require treatment (6). Unfortunately, the decision to place a patient on AS, as well as determining when surveillance may no longer be appropriate, remains controversial due to limitations in both risk stratification and disease monitoring (7, 8). In fact, current AS inclusion criteria may misclassify as many as one in four patients (9, 10). Moreover, studies of AS-eligible men who opted for early radical prostatectomy were found to have significant cancer in up to 20% of cases (7, 11). Therefore, optimization of diagnostic and surveillance strategies are necessary to minimize patient risk.

Over the past several years, the use of multiparametric MRI has increased dramatically for the initial diagnosis and monitoring of PCa (12, 13). In addition, the emergence of novel PCa biomarkers are increasingly available for clinical use (14). However, most well-established AS protocols have yet to formally incorporate these diagnostic modalities. We performed a review of recently published literature to characterize the emerging evidence in support of advanced imaging and genetic and epigenetic screening techniques for monitoring patients on AS for localized PCa.

## Materials and Methods

An English literature search was conducted on PubMed for available original investigations on localized PCa, AS, imaging and biomarkers. All articles published within the past few years (January 1, 2014 to August 4, 2017) were considered. Our Boolean criteria included the following terms: PCa, AS, imaging, biomarker, genetic, genomic, prospective, retrospective, and comparative. Our search excluded publication types such as comments, editorials, guidelines, reviews, or interviews. The bibliographies and diagnostic modalities of the identified studies were used to expand our search.

## Results

**Figure 1:**
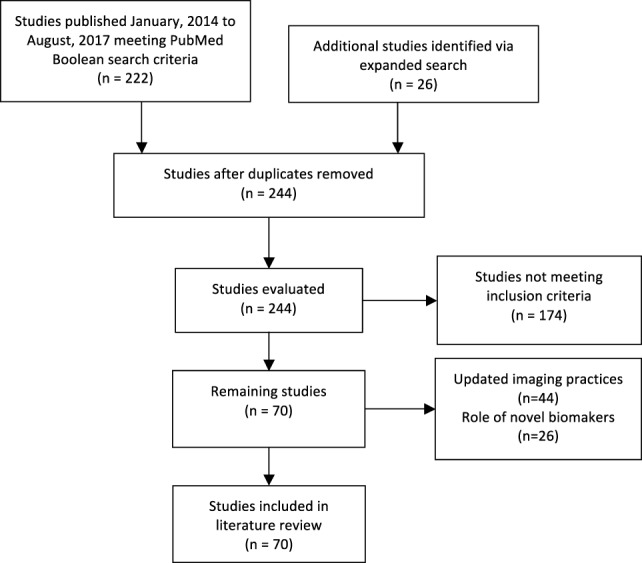
Flow chart of article selection following PubMed search.

Our review identified 222 original studies. Our expanded search yielded 244 studies. Among these, 70 publications met our inclusion criteria. A total of 44 studies assessed the role for updated imaging practices such as multiparametric magnetic resonance imaging (mpMRI), and 26 evaluated the reliability of novel biomarkers in AS cohorts (Figure 1) (15).

### Incorporation of MRI

#### Improvements in MRI Technique

In recent years, mpMRI has emerged as a reliable tool for monitoring patients on AS (13, 16). The implementation of novel MRI analytic techniques such as diffusion-weighted imaging (DWI) and volumetric estimation algorithms have allowed for improved tumor characterization (17–19). More specifically, the apparent diffusion coefficient, which is calculated from DWI sequences, inversely correlates with PCa aggressiveness (18). Furthermore, automated calculations of lesion volume on mpMRI reliably correspond with PCa presence (18, 20). Marin et al. demonstrated that the use of semi-automated sizing algorithms to measure maximal tumor dimensions of suspicious lesions reliably correlated with actual tumor histologic diameter, and this practice should be considered during routine workup (20).

#### Improved Detection of Significant PCa

Several studies suggest using mpMRI in the initial diagnostic setting may classify patients as AS-eligible more reliably than traditional protocol criteria alone (21–23). In a study by Ouzzane et al., 10% of patients initially classified as appropriate for AS were reclassified as inappropriate following the detection of clinically silent lesions on mpMRI followed by targeted biopsy (22). When reviewed by an experienced radiologist, Porpiglia et al. suggests use of mpMRI alone may reliably predict pathologically significant PCa without confirmatory tissue biopsy (21). However, in a retrospective study of 118 patients, Dianat et al. found significant pathology could exist in the absence of a visible index lesion on MRI (24).

#### Optimization of AS Protocols by Incorporating Prostate Imaging Reporting and Data System (PIRADS)

Over the past 10 years, the PIRADS has become an increasingly useful tool in both the initial diagnostic setting and for monitoring patients on AS (25–27). The PIRADS system is a five-point Likert scale applied to a suspicious lesion on MRI. An overall score is assigned to predict the likelihood that a suspicious lesion harbors clinically significant PCa (28, 29). In a retrospective analysis of more than 1,000 patients on AS, Venderink et al. demonstrated that a PIRADS cutoff of ≥3 could more reliably select patients for repeat biopsy than PSA density (PSAD) (26). In a study of 201 patients undergoing transperineal sector prostate biopsy, Grey et al. demonstrated that a strict cutoff of PI-RADS ≥3 would have allowed 44% of men to avoid undergoing repeat biopsy (25). However, 2.3% of those men still harbored significant PCa, raising the question as to what degree of specificity is necessary for proper management of patients on AS with presumably slow-growing disease (25). We identified three original investigations that compared a widely accepted AS protocol with a predictive nomogram incorporating PIRADS, and all three studies demonstrated a significant improvement in risk stratification (30–32).

More recently, a proposed PIRADS version 2 (PIRADSv2) was developed to reflect increasingly complex MRI interpretation with the application of multiple sequences such as DWI and dynamic contrast enhancement for interpretation of a single lesion (27, 29, 33). Validation studies comparing its utility to the original PIRADS algorithm are currently underway. However, early results indicate significant PCa may be overlooked as often as 5% of the time when using PIRADSv2 ≥3 as a cutoff (27). Furthermore, there is concern that the PIRADS algorithm may inaccurately characterize benign central zone (CZ) lesions. In a retrospective review of 73 patients who underwent MRI-fusion biopsy by Tan et al., 26 CZ lesions were graded as PIRADS 3 or greater. Only two (7.7%) of these suspicious lesions harbored PCa (34). Since evidence suggests PCa arising from the CZ can be more aggressive than peripheral and transitional zone cancers, modification of the PIRADS algorithm may be needed to better characterize CZ lesions (35, 36).

#### Improved Surveillance Biopsy Technique with MRI-Targeting

With mpMRI becoming an increasingly reliable predictor of histologic tumor burden, MRI–US fusion technology has been developed to perform targeted biopsy of suspicious prostatic lesions (37, 38). Several recently published investigations demonstrate performing targeted biopsies on men undergoing AS is superior to standard TRUS biopsy for the detection of newly significant PCa (39–42). Furthermore, Siddiqui et al. suggest performing MRI–US fusion targeted biopsy may reduce the number of insignificant PCa diagnoses, thus sparing patients from unnecessary biopsies (43).

However, there is conflicting data whether it is appropriate to only use MRI–US fusion biopsy, thus abandoning initial random TRUS guided sampling, for the detection of Gleason score (GS) upgrades in AS patients (39–41, 44). For example, Da Rosa et al. reported a 100% negative predictive value for detecting GS 6 to 7 upgrade when using MRI–US fusion in their cohort of 72 men on AS (40). Furthermore, a retrospective study by Nassiri et al. of more than 250 patients showed that 32 of 33 upgraded cases were from MRI-guided cores (41). In contrast, a study by Marliere et al., albeit with a smaller cohort, demonstrated both standard TRUS and targeted MRI–US fusion biopsies in the initial AS setting may still be necessary for adequate detection of all new GS upgrades (44).

Compared to standard TRUS sampling and MRI–US fusion targeted biopsy, evidence demonstrates saturation biopsy (24 or 30 core templated sampling) provides the greatest sensitivity for detection of significant PCa in the initial AS period (45–47). While this approach may provide enhanced detection, it also subjects patients to the burden of increased tissue acquisition. Pepe et al. proposed using a hybrid approach they referred to as “cognitive zonal fusion biopsy” (46). In this approach, all patients undergo mpMRI prior to a MRI–US fusion biopsy. If during the biopsy there is a discrepancy between what was found on MRI and what is visualized on US, two to six cores are obtained from the MRI region of interest. In their prospective study of 58 individuals who were either biopsy naïve or on AS, this approach reliably detected significant PCa (46).

#### mpMRI May Allow for Biopsy-Free Surveillance Protocols

There is mounting evidence that men on AS who underwent proper initial evaluation may be monitored with serial mpMRIs, and the interval for repeat biopsies may be lengthened (8, 48–52). In two separate studies of men on AS who underwent serial mpMRIs and MRI–US fusion biopsy, both Walton Diaz et al. and Felker et al. observed that stable findings on mpMRI significantly correlated with GS stability (48, 50). In a 2-year retrospective review of 162 men on AS, Frye et al. demonstrated that progression on repeat mpMRI significantly predicted pathological progression (8).

For men with small index lesions (≤7 mm), a retrospective review of more than 150 patients on AS demonstrated no change in either lesion size or pathologic characteristics during a 2-year surveillance period (51). This suggests that men on AS, whose lesions meet this size criteria, could potentially forgo any surveillance whatsoever for intervals up to 2 years. Siddiqui et al. developed a predictive nomogram based on serial mpMRI results for men on AS who underwent repeat MRI–US fusion biopsies over the course of 5 years. Based on whether targeted biopsy prompted disqualification from AS, their nomogram incorporating mpMRI could have potentially avoided repeat tissue biopsy sessions in up to 68% of men (49).

### Role of Biomarkers

Recent advances in genomic sequencing and molecular classification led to development of a plethora of assays for PCa diagnosis. Unquestionably, serum PSA is the most frequently used biomarker for detecting and monitoring PCa. However, there are several well-documented limitations in its reliability for predicting disease presence (14). Benign prostatic diseases, DRE, urologic instrumentation, and recent ejaculation may all cause the serum PSA to become elevated in absence of PCa (14, 53). Therefore, considerable effort has been given to identify novel tissue, serum, and urine-based biomarkers to better stratify at-risk patients.

#### 4K Score

Kallikrein-related enzymes are a family of serine proteases with high homology (54). While the kallikrein-3 gene (KLK-3, or PSA) is among the most extensively investigated in relation to PCa, expression of all 15 kallikreins can be detected in prostate tissue (55). Recently a predictive tool comprising plasma total PSA, free PSA, intact PSA, and kallikrein-2 along with patient age, DRE and biopsy history known as the 4Kscore Test has gained attention as a novel risk stratification tool for patients with elevated PSA, and possibly newly diagnosed PCa (55–57). In a prospective study by the Canary Prostate Active Surveillance Study Investigators, which evaluated more than 700 men with newly diagnosed Gleason 6 PCa on AS, the 4Kscore reliably predicted GS upgrades on the first surveillance biopsy (57). However, there was a decreased utility in the 4Kscore Test for predicting GS upgrades on subsequent biopsies, suggesting it may only be beneficial in the initial AS period (57).

#### SelectMDx and ConfirmMDx

A novel urinary assay-based risk score called SelectMDx by MDxHealth combines serum PSA, PSAD and clinical factors such as age and prior negative biopsy with two mRNA signatures: urinary homeobox C6 and distal-less homeobox 1 (58). This relatively low-cost assay was recently implicated as a useful diagnostic aid for appropriately selecting men for undergoing prostate biopsy (58). In the setting of a prior negative biopsy, MDxHealth developed an additional assay called ConfirmMDx (59, 60). The ConfirmMDx assay utilizes methylation analyses of various oncogenes such as Ras and Adenomatous Polyposis Coli from a patient’s prior negative biopsy tissue to estimate the likelihood of obtaining a negative repeat biopsy (59). In a multicenter study of 350 subjects, Partin et al. reported an 88% negative predictive value of detecting significant PCa at 13-month follow-up. Furthermore, their multivariate model suggested ConfirmMDx may independently predict repeat negative biopsy in this setting (60).

#### Prolaris

The Prolaris test evaluates the RNA expression of 46 genes to evaluate tumor cell growth characteristics and may help risk stratify patients with PCa (61–63). The assay measures the cell cycle progression score, and has proven prognostic value in other malignancies such as breast cancer (64). Several recent investigations revealed performing the Prolaris test in the initial diagnostic setting may reliably risk stratify patients for AS, and potentially avoid unnecessary repeat biopsies for those with low risk Prolaris scores (61, 65).

#### Oncotype Dx

The Oncotype Dx panel is a 17-gene reverse transcription polymerase chain reaction-based diagnostic assay that has been validated as a predictor of adverse pathology following prostatectomy in men with low-risk PCa (66–69). Eure et al. prospectively studied 297 patients on AS, and those who underwent Oncotype Dx screening were more likely to remain on AS (66). The authors suggest the predictive value of Oncotype Dx may provide both patients and clinicians with better peace of mind when opting to remain on AS given additional reassurance that AS is safe given a indolent genetic profile of disease.

#### Decipher

The Decipher gene expression assay uses 22 genes to predict the risk of developing metastases following radical prostatectomy (70, 71). While there is evidence supporting its utility in the postoperative setting, we could not identify any studies evaluating the potential benefit of using the Decipher assay for AS patients undergoing conservative management.

#### ProMark

Metamark Genetics Inc. developed a quantitative protein-based multiplex imaging platform designated ProMark as prognostic test for PCa. ProMark’s quantitative immunofluorescence method utilizes imaging platform and measure eight biomarkers directly on sections of biopsy tissue (72). Multiple studies have tested the utility of the ProMark assay in predicting biochemical recurrence after radical prostatectomy (73, 74).

#### PTEN

The phosphatase and tensin homolog (PTEN) is a tumor suppressor gene whose function may be lost in patients with PCa (75). As such, measuring PTEN to predict PCa severity has gained attention as a possible way to stratify patients on AS (76, 77). In a retrospective study using tissue microarray specimens of over 600 patients who underwent radical prostatectomy, loss of PTEN was significantly correlated with PCa severity (76). Lokman et al. recently demonstrated that loss of PTEN on pathological analysis of surveillance targeted biopsies may predict future GS upgrading and AS discontinuation (76).

#### ERG

Majority of PCa show recurrent chromosomal rearrangement which leads predominantly to the fusion of the androgen-responsive promoter elements of the TMPRSS2 gene with ETS transcription factor ERG. This gene fusion leads to overexpression of ERG in PCa (78). In a study of 265 patients on AS, Berg et al. demonstrated ERG positivity was significantly associated with progression of disease (79). Furthermore, a separate study by Berg et al. showed ERG expression remains consistent from initial biopsy through surgical removal of the prostate (80). This temporal stability suggests clinicians could obtain ERG expression just one time at any point in a patient’s disease course, thus limiting the costs of re-sampling.

#### Prostate Health Index (PHI) and Prostate Cancer Antigen-3 (PCA3)

The PHI is a formula that incorporates the following PSA isoforms: total PSA, free PSA, and pro-PSA (81). PCA3 is a gene that expresses noncoding RNA which is extracted from the prostate following DRE (81, 82). Both assays have gained attention as potential tools for AS patient selection and monitoring (81–84). Recently, Cantiello et al. demonstrated both PHI and PCA3 may aid in the prediction pathologically insignificant PCa (84). However, this was a retrospective observational study, and few if any studies have investigated the predictive value of these biomarkers in a prospective clinical setting (81, 84).

#### CXCL12

The α-chemokine receptor, C–X–C chemokine receptor type 4 (CXCR4) and its ligand, stromal-derived-factor 1 (also known as CXCL12) are therapeutic targets in various epithelial, mesenchymal, and hematopoietic tumors (85, 86). Recent evidence suggests the CXCR4/CXCL12 interaction may be involved in PCa tumorigenesis (87, 88). In a retrospective review of patients who underwent radical prostatectomy, Goltz et al. demonstrated that aberrant CXCL12 methylation was correlated with GS and biochemical recurrence (89). They postulated that evaluating CXCL12 in the AS setting may be beneficial, but we failed to identify any studies to date that have evaluated this theory.

#### CPCs

The detection of malignant circulating prostate cells (mCPCs) has gained attention as a novel, minimally invasive way to detect PCa status (90–92). By using immunocytochemistry and anti-PSA monoclonal antibodies, detection of plasma mCPCs may reliably identify low vs high risk PCa (92). In a retrospective study of 161 men on AS, Murray et al. demonstrated mCPC detection was superior to both the Chun nomogram and serum-free PSA for predicting the detection of clinically significant PCa (91). Using mCPC presence alone resulted in only two (<1%) missed PCa diagnoses, and both were low-grade disease.

#### Single-Nucleotide Polymorphisms (SNPs)

Genome-wide association studies have implicated numerous SNPs in PCa development (93, 94). There is considerable interest in identifying SNPs associated with high risk PCa to aid in identifying patients eligible for AS (95). Recently, Kearns et al. identified a SNP associated with GS upgrading in a cohort of more than 200 patients on AS (96). Their findings warrant further investigation of the predictive insight SNPs may provide.

## Discussion

Active surveillance is a widely adopted strategy to monitor patients with low-risk, localized PCa, and offers both cost and quality of life benefits to patients meeting surveillance criteria. Optimally monitoring men on AS remains a challenge for clinicians due to disease variability, the lack of an overall consensus on optimal management, and the abundance of readily available diagnostic tests. A majority of AS protocols use data obtained from DREs, PSAs, and TRUS biopsies to stratify patients (3–5). Since up to 50% of all patients on AS will eventually require treatment of their cancer, and evidence suggests that some men are inappropriately placed on surveillance, improved diagnostic modalities are needed (6–11). The advent of improved MRI techniques and our expanding knowledge of genetic alterations driving PCa tumorigenesis have facilitated the development of novel diagnostic strategies for monitoring PCa.

Recent evidence suggests using mpMRI to characterize suspicious lesions is superior to currently accepted strategies, and doing so may avoid unnecessary repeat biopsies in a significant number of men (17–19, 21–23). Moreover, when used in the initial diagnostic setting, mpMRI, may actually identify more aggressive disease not found on the initial biopsy and, therefore, may keep the patient from being inappropriately placed on AS (22). Recently, the European School of Oncology formed the PCa Radiological Estimation of Change in Sequential Evaluation (PRECISE) panel to develop a consensus on mpMRI data reporting for researchers investigating cohorts of men on AS. While validation of the panel’s initial recommendations is needed, this may aid in future optimization of currently used diagnostic nomograms (52).

When prostate biopsy is indicated, the integration of MRI technology with TRUS biopsy *via* MRI–US fusion technology enhances the ability to sample concerning lesions with pinpoint accuracy (39–42). Recent evidence suggests that performing MRI-targeted biopsies may be just as reliable as saturation biopsies for detection of significant PCa (45–47). This would spare both patients and providers the burden of acquiring unnecessary cores, and may lessen the frequency of detecting insignificant disease (46). However, some studies suggest MRI–US fusion alone may not be adequate in the initial diagnostic setting, and standard template biopsy may be equally diagnostic in biopsy naïve men (44).

While PSA is the most commonly used serum biomarker to detect and monitor PCa, many novel genetic and epigenetic markers of PCa disease status are under investigation. Several commercially available screening panels exist, and have promise in risk stratification of men newly diagnosed with PCa (55–57, 61, 65–70). Among these, our review found some evidence supporting the use of the 4K Score, SelectMDx, ConfirmMDx Prolaris, and Oncotype Dx in both the initial diagnostic and AS milieus (57, 58, 60, 61, 65, 66).

Novel biomarkers such as PTEN, ERG, PCA-3, CXCL12 and utilization of various PSA isoforms are gaining attention as potential indicators of PCa disease (76, 79, 84, 89). Indirectly assessing PCa status in the form of both mCPC and SNP detection has also been proposed for patients in the AS setting (91, 96). While these candidate markers show promise, they are by and large still in their nascent stage of development, and further validation in a diverse prospective setting is warranted. Furthermore, the potential logistical and financial burden of performing large prospective studies using these advanced diagnostic panels should not be overlooked.

## Conclusion

In the era of AS for men with low-risk PCa, improved strategies for proper stratification are needed to balance overtreatment with under diagnosis. mpMRI has dramatically enhanced the detection of clinically significant PCa, and may permit less-invasive surveillance strategies compared to currently accepted protocols. The advent of novel biomarkers for prediction of aggressive disease and AS failure has shown some initial promise, but further validation is warranted.

## Author Contributions

ZG: data acquisition, data processing, and drafting and critical review of manuscript. JG: data processing, and drafting and critical review of manuscript. KP: data processing, and drafting and critical review of manuscript. SV: data processing, drafting and critical review of manuscript. SR-B: idea development, data acquisition, data processing, drafting and critical review of manuscript.

## Conflict of Interest Statement

The authors declare that the research was conducted in the absence of any commercial or financial relationships that could be construed as a potential conflict of interest.

## Abbreviations

ADC, apparent diffusion coefficient; AS, active surveillance; CCP, cell cycle progression score; CZ, central zone; DWI, diffusion-weighted imaging; GS, Gleason score; mpMRI, multi parametric magnetic resonance imaging; MRI, magnetic resonance imaging; PCa, prostate cancer; PHI, prostate health index; PIRADS, Prostate Imaging Reporting and Data System; PSA, prostate specific antigen; PSAD, PSA density; TRUS, transrectal ultrasound.
